# Breadth and Durability of SARS-CoV-2-Specific T Cell Responses following Long-Term Recovery from COVID-19

**DOI:** 10.1128/spectrum.02143-23

**Published:** 2023-07-10

**Authors:** Thi Thu Thao Dang, Alitzel Anzurez, Kaori Nakayama-Hosoya, Shoji Miki, Kazuo Yamashita, Mark de Souza, Tetsuro Matano, Ai Kawana-Tachikawa

**Affiliations:** a AIDS Research Center, National Institute of Infectious Diseases, Tokyo, Japan; b Joint Research Center for Human Retrovirus Infection, Kumamoto University, Kumamoto, Japan; c KOTAI Biotechnologies, Inc., Suita, Japan; d Department of AIDS Vaccine Development, Institute of Medical Science, University of Tokyo, Tokyo, Japan; Kumamoto Daigaku

**Keywords:** SARS-CoV-2, COVID-19, T cell immunity, variants

## Abstract

T cell immunity is crucial for long-term immunological memory, but the profile of severe acute respiratory syndrome coronavirus 2 (SARS-CoV-2)-specific memory T cells in individuals who recovered from COVID-19 (COVID-19-convalescent individuals) is not sufficiently assessed. In this study, the breadth and magnitude of SARS-CoV-2-specific T cell responses were determined in COVID-19-convalescent individuals in Japan. Memory T cells against SARS-CoV-2 were detected in all convalescent individuals, and those with more severe disease exhibited a broader T cell response relative to cases with mild symptoms. Comprehensive screening of T cell responses at the peptide level was conducted for spike (S) and nucleocapsid (N) proteins, and regions frequently targeted by T cells were identified. Multiple regions in S and N proteins were targeted by memory T cells, with median numbers of target regions of 13 and 4, respectively. A maximum of 47 regions were recognized by memory T cells for an individual. These data indicate that SARS-CoV-2-convalescent individuals maintain a substantial breadth of memory T cells for at least several months following infection. Broader SARS-CoV-2-specific CD4^+^ T cell responses, relative to CD8^+^ T cell responses, were observed for the S but not the N protein, suggesting that antigen presentation is different between viral proteins. The binding affinity of predicted CD8^+^ T cell epitopes to HLA class I molecules in these regions was preserved for the Delta variant and at 94 to 96% for SARS-CoV-2 Omicron subvariants, suggesting that the amino acid changes in these variants do not have a major impact on antigen presentation to SARS-CoV-2-specific CD8^+^ T cells.

**IMPORTANCE** RNA viruses, including SARS-CoV-2, evade host immune responses through mutations. As broader T cell responses against multiple viral proteins could minimize the impact of each single amino acid mutation, the breadth of memory T cells would be one essential parameter for effective protection. In this study, breadth of memory T cells to S and N proteins was assessed in COVID-19-convalescent individuals. While broad T cell responses were induced against both proteins, the ratio of N to S proteins for breadth of T cell responses was significantly higher in milder cases. The breadth of CD4^+^ and CD8^+^ T cell responses was also significantly different between S and N proteins, suggesting different contributions of N and S protein-specific T cells for COVID-19 control. Most CD8^+^ T cell epitopes in the immunodominant regions maintained their HLA binding to SARS-CoV-2 Omicron subvariants. Our study provides insights into understanding the protective efficacy of SARS-CoV-2-specific memory T cells against reinfection.

## INTRODUCTION

Tcell immunity plays a critical role in controlling viral infection and immunological memory. Multiple studies have been conducted on T cell responses to severe acute respiratory syndrome coronavirus 2 (SARS-CoV-2) using blood samples from COVID-19 patients or individuals recovering from COVID-19 and suggest the importance of T cell responses in protective immunity to COVID-19 (1–6).

The quality of T cell immunity is generally represented by the frequency of specific T cells and their functions, e.g., cytokine production, cytotoxicity, and proliferative capacity, whereas the breadth of T cell responses is also a critical parameter. While neutralizing antibodies target a limited array of proteins that block virus entry, T cells can recognize peptides derived from multiple viral proteins and directly kill virus-infected cells. Following SARS-CoV-2 infection, T cell responses are induced to multiple targets across the virus proteome (5, 7–9).

A single amino acid substitution within an epitope is sufficient for complete evasion from a T cell response, and escape from T cell responses is associated with disease progression in infections caused by viruses with high mutation rates (10–13). SARS-CoV-2 variants have emerged continuously throughout the pandemic and most possess escape mutations from neutralizing antibodies (14–18), a major concern due to the reduction of efficacy of preexisting or vaccine-induced antibodies. However, the role of amino acid substitutions in SARS-CoV-2 variants in viral escape from memory T cells remains unclear. Therefore, it is crucial to identify individual regions that are recognized by T cells and to assess how amino acid mutations in these regions affect cognate T cell responses.

Although several studies have performed identification of SARS-CoV-2 immunodominant regions, most studies focused on epitopes presented by prevalent HLA molecules for a specific geographic region (7, 19–31), with a limited number of studies performing a comprehensive analysis (5, 9, 32). One assessment of SARS-CoV-2-specific T cells across the entire viral proteome using peripheral blood mononuclear cells (PBMCs) was performed in North America (9), with complete mapping of CD4^+^ T cell responses to spike (S), nucleocapsid (N), and membrane (M) proteins. However, analysis of CD8^+^ T cell responses focused on epitopes presented by common HLAs for that region.

Identification of antigenic regions eliciting T cell responses requires large numbers of PBMCs; hence, blood volume collection limitations pose a major challenge. Furthermore, virus-specific T cells contract following clearance of viral antigens, with a relatively small number maintained as memory against reinfection. This reduced memory T cell population in peripheral blood may underestimate a single peptide-specific response because of their low frequency or being masked by background responses in assays frequently used for such analyses, including enzyme-linked immunosorbent spot assay (ELISpot) and flow cytometry. In the present study, PBMCs from Japanese individuals who recovered from COVID-19 (COVID-19-convalescent individuals) were expanded with SARS-CoV-2 antigens *in vitro* to acquire higher numbers of SARS-CoV-2-specific T cells and increase the sensitivity of our assays. Comprehensive screening of T cell responses to S and N proteins was performed using *in vitro*-expanded PBMCs. Regions frequently recognized by memory T cells were identified, and the impact of amino acid mutations in these regions on T cell responses was assessed for SARS-CoV-2 variants.

## RESULTS

### Assessment of SARS-CoV-2-specific memory T cells with *ex vivo* PBMCs.

SARS-CoV-2-specific T cell responses were first measured with an interferon gamma (IFN-γ) ELISpot assay using PBMCs from 38 individuals who recovered from COVID-19 (Table 1). The distribution of HLA class I alleles was equivalent to that in the general Japanese population (33) (see Fig. S1 in the supplemental material). The maximum numbers of days post-symptom onset (DPSO) of blood sampling were 313 and 299 for the severe and mild cases, respectively.

**TABLE 1:** Participant characteristics

Parameter	Value for group			*P* value
	All	Severe	Mild	
No. of patients	38	10	28	
Sex, no. (%)				
Male	26 (68.4)	6 (60)	20 (71.4)	
Female	12 (31.6)	4 (40)	8 (28.6)	
Age (yrs)	45 (IQR, 32–53.5)	44 (IQR, 37–52)	47 (IQR, 29–54)	
DPSO^*a*^ at sampling	135 (IQR, 90–219)	257 (IQR, 111.5–297)	129.5 (IQR, 86.5–183)	0.0314

a Days post-symptom onset.

**FIG 1:**
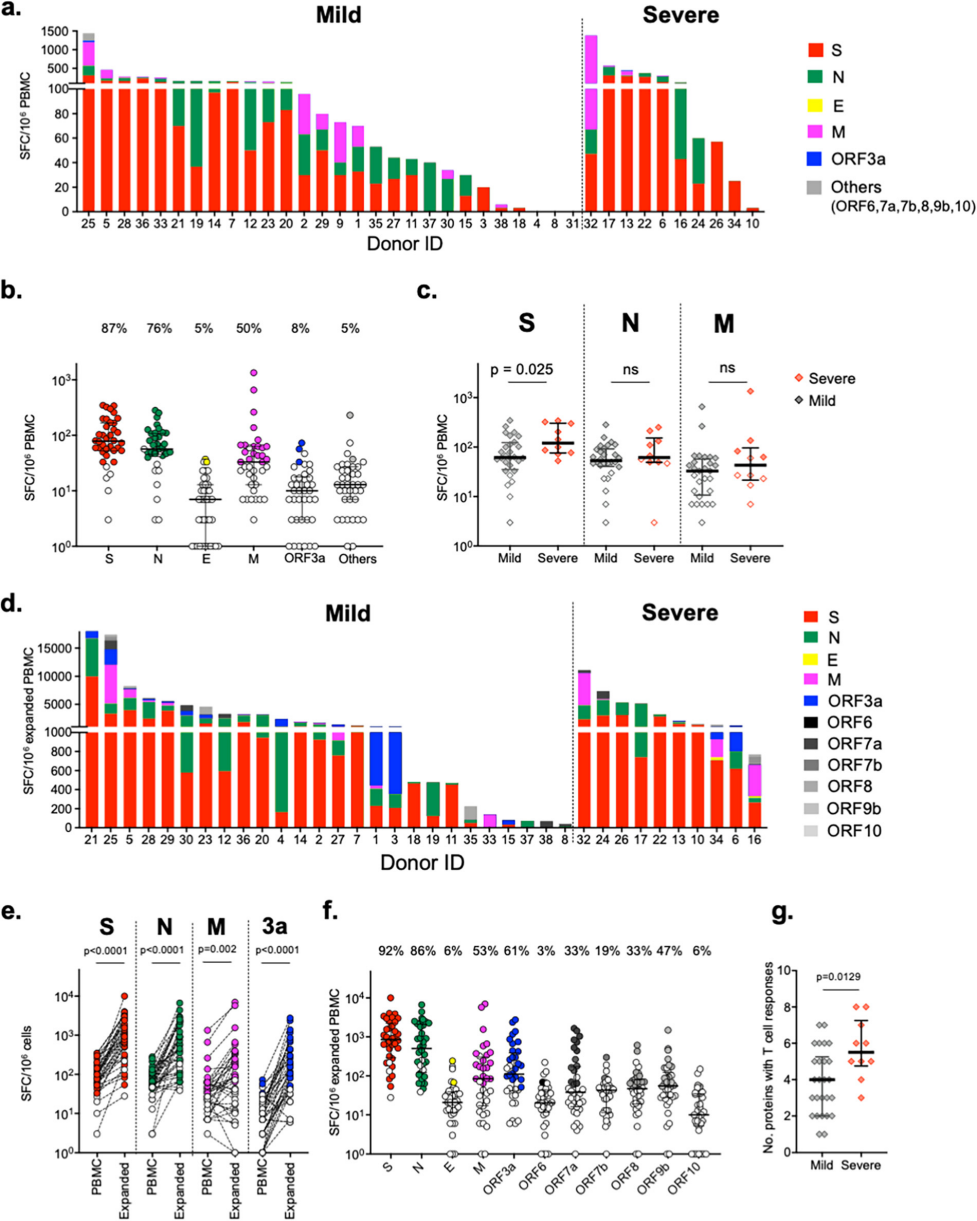
Magnitude of SARS-CoV-2-specific memory T cells in COVID-19-convalescent individuals. T cell responses against SARS-CoV-2 proteins in PBMCs (a to c) and *in vitro* expanded PBMCs (d to g) were measured by the IFN-γ ELISpot assay. Bars show the total T cell responses specific to all SARS-CoV-2 proteins tested for each individual. (a and d) Corrected (minus the negative control) SFC/10^6^ PBMCs. (b and f) Magnitude (SFC/10^6^ PBMCs) and frequency (percent) of T cell responses against each protein. (c) Magnitude (SFC/10^6^ PBMCs) of T cell responses specific for the indicated proteins between groups with mild and severe COVID-19. (e) Magnitude of T cell-specific responses to SARS-CoV-2 proteins before (PBMC) and after (Expanded) *in vitro* expansion. (g) Number of proteins recognized by memory T cells for the 11 SARS-CoV-2 proteins tested in the mild and the severe groups. Negative responses are shown as open circles in panels b, c, e, and f. Differences between groups were determined using the paired *t* test (*ex vivo* versus expanded PBMCs) and the Mann-Whitney test. ns, not significant.

Due to cell number limitations, overlapping peptide (OLP) pools of selected SARS-CoV-2 proteins (ORF6, ORF7a, ORF7b, ORF8, ORF9b, and ORF10) were combined for the ELISpot assay. T cell responses against at least a single viral protein were detected in 35 of 38 (92%) individuals. The magnitude of T cell responses varied considerably between individuals (Fig. 1a). The highest frequency of T cell responses was to the S antigen (33/38 individuals [87%]), followed by the N (76%) and M (50%) proteins (Fig. 1b). The magnitude of T cell responses was highest to the S protein (median, 78 spot-forming cells [SFC]/million PBMCs; interquartile range [IQR], 52 to 168), followed by the N (median, 57 SFC/million PBMCs; IQR, 46 to 108) and M (median, 33 SFC/million PBMCs; IQR, 13 to 63) proteins. The magnitude of S protein-specific T cells, but not T cells specific to N or M protein, was significantly higher in individuals with severe symptoms (severe group) than those with mild symptoms (mild group) (*P* = 0.025) (Fig. 1c). The median number of proteins recognized was 2 for both the mild and severe groups (data not shown).

### Assessment of SARS-CoV-2-specific memory T cells with *in vitro*-expanded PBMCs.

PBMCs were cultured with SARS-CoV-2 antigens in the presence of interleukin 2 (IL-2) for 2 weeks to expand SARS-CoV-2-specific T cells. The average total number of expanded PBMCs increased about 9-fold after 2 weeks of culture (data not shown). T cell responses against all tested SARS-CoV-2 proteins were also assessed using expanded PBMCs (Fig. 1d). The magnitude of S (median, 864 SFC/million PBMCs; IQR, 289 to 2,279) and N (median, 505 SFC/million PBMCs; IQR, 124 to 2,130) protein-specific T cells after expansion was significantly correlated with that of *ex vivo* PBMCs (Fig. S2), and both were significantly higher (approximately 10-fold) than that of PBMCs (*P* < 0.0001) (Fig. 1e and f). T cells specific for other SARS-CoV-2 proteins were also detected in the expanded PBMCs, but with considerably higher frequencies than *ex vivo* PBMCs. Interestingly, while the frequency of T cell responses against E and M proteins remained similar following *in vitro* PBMC expansion, T cell responses to ORF3a protein were detected in 22 individuals (61%), compared to only *ex vivo* PBMCs from 3 individuals (8%) (Fig. 1e). These data suggest that the overall sensitivity of detection of T cell response is higher when expanded PBMCs are used in this assay. Each donor had T cell responses to multiple viral proteins in the expanded PBMCs, with a median of 4.5 proteins per individual (Fig. 1d). These results indicate that memory T cells against SARS-CoV-2 proteins possess proliferative capacity to antigen stimulation and are maintained in individuals who have recovered from COVID-19 for at least several months. Although no significant difference was found in the magnitude of T cell responses against each protein between COVID-19 severity (data not shown), unlike the finding with *ex vivo* PBMCs, the number of proteins recognized by T cells was significantly higher in the severe group than in the mild group (Fig. 1g).

### Comprehensive identification of T cell target regions in S and N proteins.

To evaluate the breadth of SARS-CoV-2-specific memory T cells, target peptides in S and N proteins were screened with two-dimensional peptide matrices using the ELISpot assay (Fig. S3).

**FIG 2:**
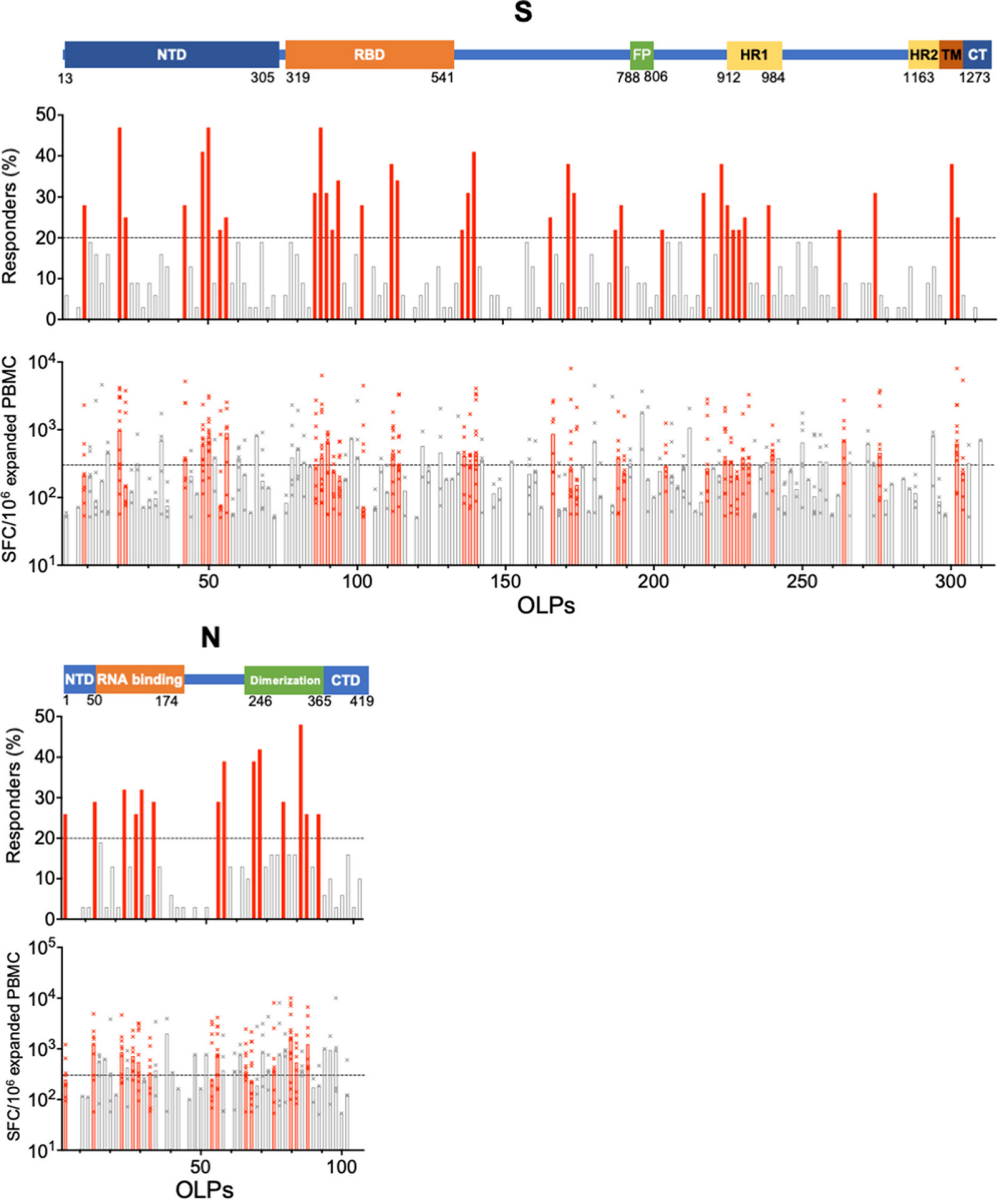
Distribution of T cell-target regions in spike (S) and nucleocapsid (N) proteins. Frequency of responders (upper graphs) and magnitude of T cells response (lower graphs) to individual overlapping peptide (OLP) pairs in SARS-CoV-2 S and N proteins are shown. The OLP pairs that recognized more than 20% of tested individuals are shown as red bars. For graphs showing frequency of responders, the black line at the *y* axis indicates the cutoff for defining regions frequently targeted by T cells (20%). For graphs showing the magnitude of the ELISpot response, the black line on the *y* axis shows the cutoff for samples selected for ICS (≥300 SFC/million expanded PBMCs). NTD, N-terminal domain; RBD, receptor binding domain; FP, fusion peptide; CT, C terminus; CTD, C-terminal domain.

**FIG 3:**
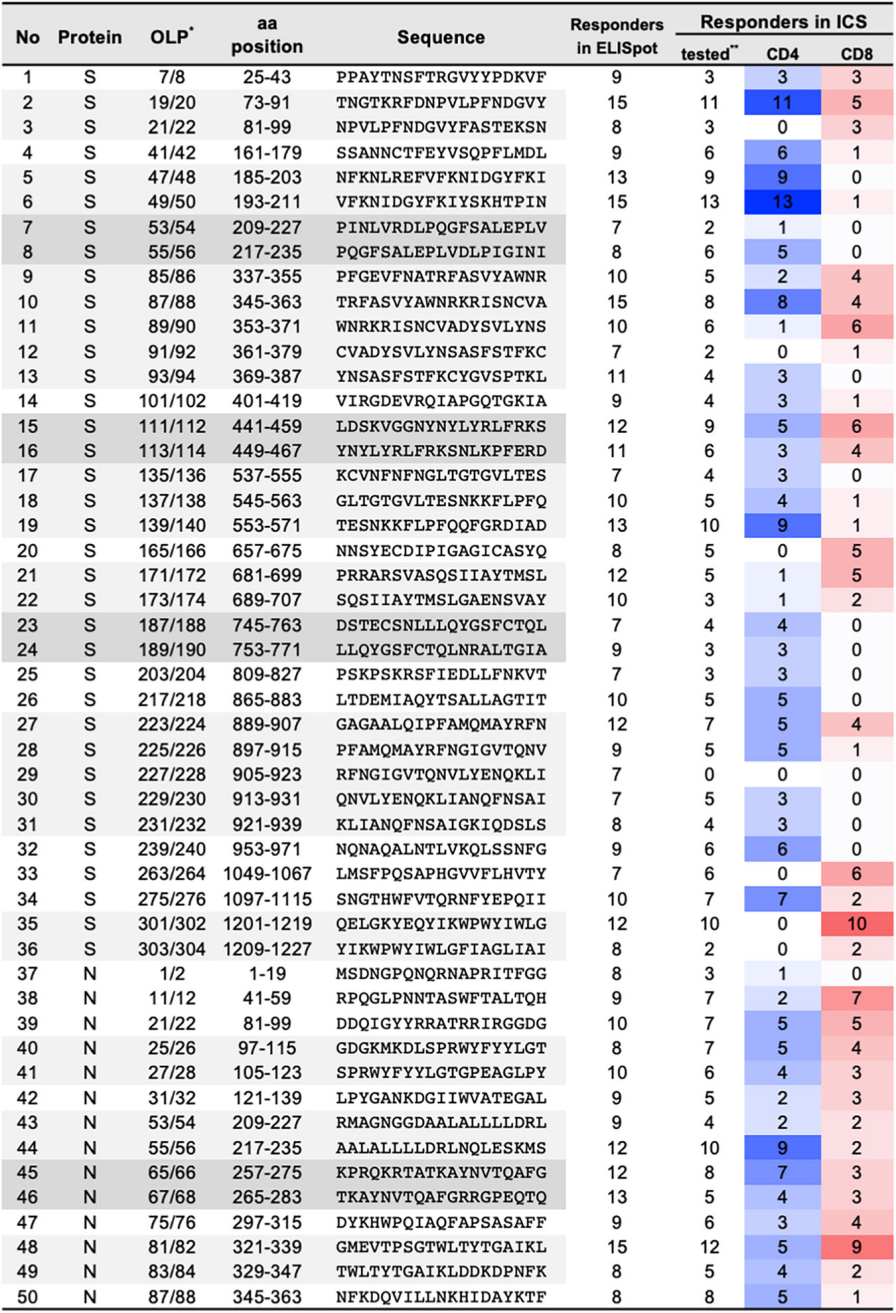
Frequent T cell target regions in spike and nucleocapsid proteins. aa, amino acid. *, overlapped peptide pairs are shown by shading. **, the numbers of the samples for which ICS was performed (the samples with ≥300 SFC/expanded PBMCs in the ELISpot assay) are shown. The frequencies of the responders with CD4^+^ and CD8^+^ T cell responses are shown as the intensities of blue and red, respectively.

T cell target peptides were successfully identified from 32 and 31 convalescent individuals for S and N proteins, respectively, using expanded PBMCs (Fig. 2). T cell target peptides were widely distributed across each protein, with 133/157 (84.7%) and 46/51 (90.2%) OLP pairs recognized by at least one individual for S and N proteins, respectively (Fig. 2). Several peptides were commonly recognized across individuals. Thirty-six and 14 OLP pairs representing 22.7% and 27.5% of S and N proteins, respectively, were recognized by T cells in more than 20% of individuals. These peptide regions were defined as frequent T cell target regions (Fig. 2 and 3).

### Evaluation of the breadth of T cell responses in S and N proteins.

**FIG 4:**
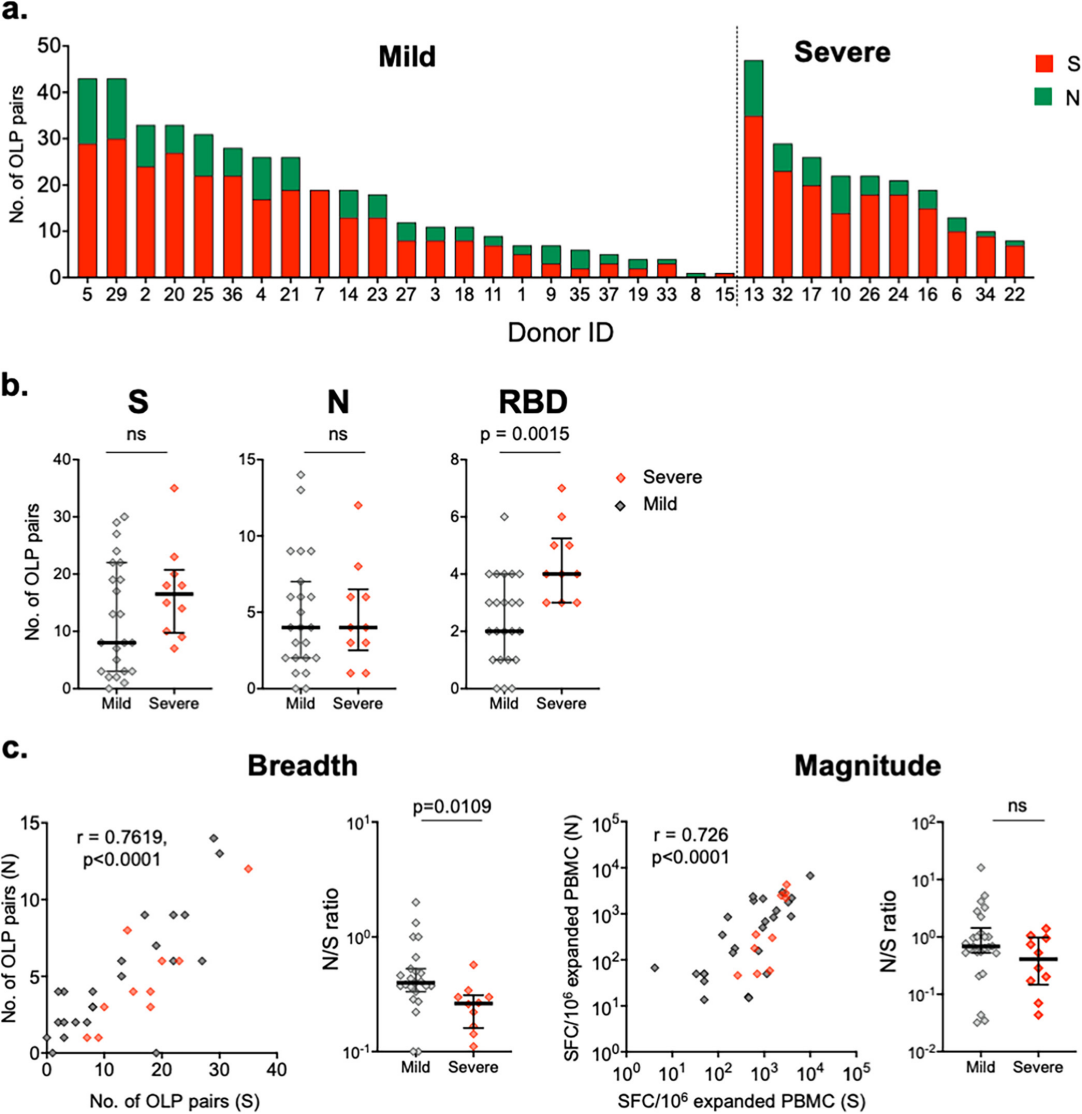
Breadth of SARS-CoV-2-specific memory T cells. (a) Number of overlapping peptide (OLP) pairs that were recognized in S and N proteins by PBMCs expanded *in vitro* from COVID-19-convalescent individuals. (b) Comparison of the breadth of memory T cells in whole S and N proteins and the RBD between the mild and severe disease groups. (c) Correlation of the breadth and magnitude of memory T cells between S and N proteins. The ratio of N protein-specific to S protein-specific T cell responses (N/S) was calculated for both the breadth and the magnitude of the T cell response. Differences between groups were determined using the Mann-Whitney test. Correlations were performed using Spearman’s test.

Measurement of the breadth of T cell responses against S and N proteins in COVID-19-convalescent individuals was performed as follows. Positive T cell responses in adjacent OLP pairs were counted as 1 (the minimum possible number), and three consecutive OLP pairs showing positive responses were scored as 2. The median breadths of T cell responses against S and N proteins were 13 (IQR, 6 to 21) and 4 (IQR, 2 to 6.5), respectively (Fig. 4a). A maximum of 47 peptides was recognized by expanded PBMCs from an individual (donor 13). The breadth of T cell responses correlated with the magnitude of the expanded PBMCs (*P* ≤ 0.0002) (Fig. S4). These data indicate that broad T cell responses were induced against both S and N proteins and maintained for up to 313 days in one individual, the maximum DPSO assessed for the study, although breadth varied considerably among the study cohort.

The median breadth of T cell responses against the S protein was higher in the severe group (16) than in the mild group (8), and the difference was significant when T cell responses against the receptor binding domain (RBD) were assessed (median, 4 for the severe group versus 2 for the mild group) (Fig. 4b). In contrast, the median breadths of T cell responses against the N protein were equivalent between groups (4 for both groups).

Significant correlations (*P* < 0.01) were observed for both the breadth and magnitude for S and N protein-specific T cell responses (Fig. 4c). The ratio of N to S proteins (N/S proteins) for breadth, but not magnitude, was significantly higher in the mild group.

### Distribution of CD4^+^ and CD8^+^ T cell responses in regions frequently targeted by T cells.

**FIG 5:**
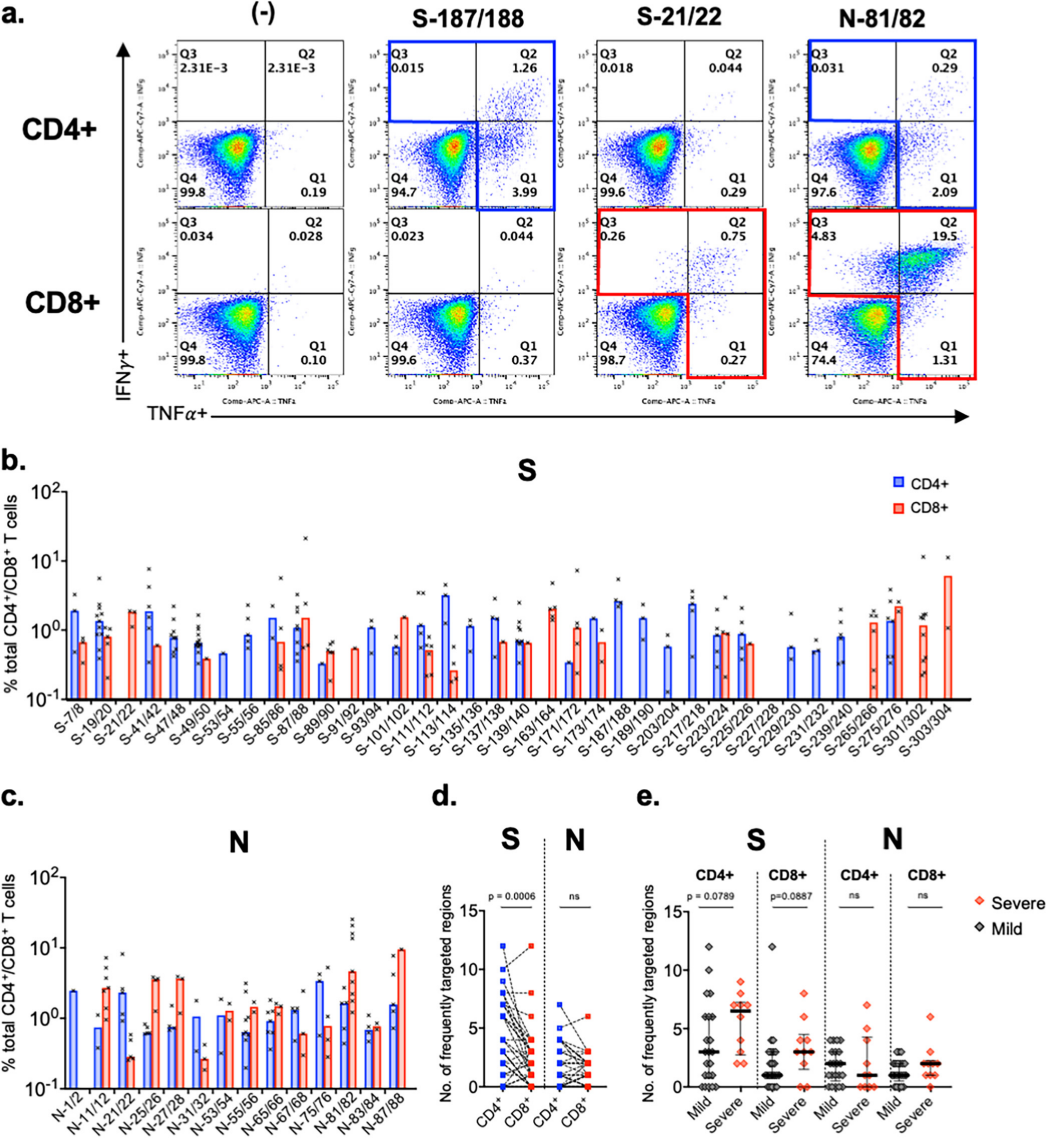
CD4^+^ and CD8^+^ T cell responses against regions of SARS-CoV-2 spike and nucleocapsid proteins frequently targeted by T cells. (a) Representative flow cytometry plots of intracellular cytokine staining (ICS) from a single individual. The T cell response against each OLP pair was defined as positive when the frequency of IFN-γ^+^ TNF-α^+^ T cells was >0.1%. (b and c) CD4^+^ and CD8^+^ T cell responses for frequently targeted SARS-CoV-2 S and N regions. ICS was performed on OLP pairs using *in vitro*-expanded PBMCs with results of >300 SFC/million cells in the ELISpot assay. The bars show the median response. (d and e) Number of SARS-CoV-2 S and N regions targeted by CD4^+^ and CD8^+^ T cells for each individual assessed. Paired *t* test (d) and Mann-Whitney U test (e) were used for comparisons.

Intracellular cytokine staining (ICS), using expanded PBMCs, was performed to elucidate the distribution of CD4^+^ or CD8^+^ T cell responses in SARS-CoV-2 regions frequently targeted by T cells (>20% of individuals assessed). Expanded PBMCs were assessed if responses of >300 SFC/million cells to OLP pairs were observed (Fig. 5). Of the 36 and 14 frequently targeted regions, positive ICS results were obtained for more than 3 individuals in 32 and all regions in S and N proteins, respectively (Fig. 2 and Fig. 5b and c). Eleven (34%) and 4 (13%) regions were recognized by CD4^+^ and CD8^+^ T cells, respectively, and 17 regions (53%) were recognized by both T cell subsets for the S protein, indicating that CD4^+^ T cell responses were dominant for the S protein (Fig. 5d). However, for the N protein, both CD4^+^ and CD8^+^ T cell responses were detected against 13 of 14 OLP pairs (93%) (Fig. 5c), showing that the two subsets equally contributed to T cell responses (Fig. 5d).

The breadths of CD4^+^ and CD8^+^ T cell responses against frequently targeted regions were compared between the severe and the mild groups, and those with severe symptoms showed a trend for broader CD4^+^ and CD8^+^ T cell responses against the S protein but not the N protein (Fig. 5e).

### Impact of amino acid mutations in SARS-CoV-2 variants on HLA binding in putative CD8^+^ T cell epitopes in regions frequently targeted by T cells.

Several SARS-CoV-2 variants have shown neutralization escape compared to the wild-type virus (Wuhan strain) (14, 15, 17, 34). Importantly, amino acid mutations that resulted in decreased neutralizing antibody sensitivity accumulated in Omicron subvariants (16, 18, 35–38), indicating that viruses that escaped from immune pressure were selected as the pandemic progressed. Therefore, we assessed how amino acid mutations in SARS-CoV-2 variants affect T cell immune responses. First, the number of amino acid changes in the S and N proteins was quantified for Delta (B.1.617.2) and Omicron subvariants (B.1.1.529, BA.1, BA.2, BA.4, BA.5, BQ.1, and XBB) in regions frequently targeted by T cells (39). While amino acid mutations in these regions were only 2 in the S protein and none in the N protein for the Delta variant, there were multiple mutations in Omicron subvariants, particularly in the S protein, where the number of amino acid mutations ranged from 10 to 17 (Table S1).

The impact of amino acid mutations on CD8^+^ T cell responses was further investigated using the epitope prediction tool NetMHCpan-4.1 (40). Epitopes presented by HLA class I alleles with a prevalence greater than 1% in the Japanese population (Fig. S1) were predicted for SARS-CoV-2 S and N protein regions frequently targeted by CD8^+^ T cells (Fig. 3). Epitope prediction was performed on 10 HLA-A, 18 HLA-B, and 11 HLA-C alleles, for which the coverages of each locus in the Japanese population are 96.85%, 93.69%, and 96.45%, respectively (33). There were 302 and 169 predicted epitopes in S and N proteins targeted by CD8^+^ T cells, respectively (Table 2 and Tables S2 and S3). There was no predicted epitope which diminished HLA binding for the Delta variant (Table 2). Although 23 to 62 (8 to 21%) of the 302 predicted epitopes in the S protein had amino acid mutations, 13 to 18 (4 to 6%) of them reduced or lost their HLA binding affinity for the Omicron subvariants assessed (Table 2). A single amino acid mutation occurring in frequently targeted CD8^+^ T cell regions in the N protein for the Omicron XBB subvariant caused a reduction or loss of HLA binding in 8 of 169 (5%) predicted epitopes.

**TABLE 2:** Amino acid mutations located in predicted CD8^+^ T cell epitopes for regions frequently targeted by T cells in SARS-CoV-2 variants

Protein	No. of predicted epitopes	Parameter	No. (%) in variant							
			Delta, B.1.617.2	Omicron						
				B.1.1.529	BA.1	BA.2	BA.4	BA.5	BQ.1	XBB
S	302	Amino acid mutations	2	8	8	9	10	10	12	15
		Epitopes with amino acid mutation	8 (3)	23 (8)	23 (8)	20 (7)	25 (8)	25 (8)	35 (12)	62 (21)
		Epitopes with less/no HLA binding^*a*^	0 (0)	13 (4)	13 (4)	13 (4)	13 (4)	13 (4)	16 (5)	18 (6)
N	169	Amino acid mutations	0	0	0	0	0	0	0	1
		Epitopes with amino acid mutation	0 (0)	0 (0)	0 (0)	0 (0)	0 (0)	0 (0)	0 (0)	14 (8)
		Epitopes with less/no HLA binding^*a*^	0 (0)	0 (0)	0 (0)	0 (0)	0 (0)	0 (0)	0 (0)	8 (5)

a Epitopes with percent rank of ≥0.5.

To further define epitopes by CD8^+^ T cells, HLA class I genotypes of individuals with positive responses and HLA restriction of predicted epitopes were assessed for each region targeted by CD8^+^ T cells. Epitopes restricted by HLA which shared by any of the responders were selected. The selected epitopes are shown (yellow highlight) in Table S2. Thirty (23%) of 129 selected epitopes possessed amino acid mutations, and only 6/129 (3%) of the mutated epitopes showed diminished HLA binding (Table S4).

These data suggest that amino acid mutations in S and N proteins of Omicron subvariants had minimal impact on the presentation of CD8^+^ T cell epitopes.

## DISCUSSION

In this study, memory T cells for SARS-CoV-2 were characterized in COVID-19-convalescent individuals. *In vitro* expansion of PBMCs with SARS-CoV-2 peptides enabled assessment of SARS-CoV-2-specific memory T cells with high resolution and sensitivity. Antigen-specific T cells were selectively expanded during the culture period, resulting in enhanced detection of low-level T cell responses. T cell responses that are unlikely to be detected in *ex vivo* PBMCs were detected in the expanded PBMCs with much higher frequency in the present study for most proteins assessed. The possibility that *in vitro* PBMC culture could expand T cells cross-reactive with SARS-CoV-2 antigens was explored using PBMCs obtained prior to the pandemic from 8 healthy individuals and analyzed under identical conditions. SARS-CoV-2-specific T cell responses were not detected in *ex vivo* PBMCs. However, low-level T cell responses were detected only against the S protein using expanded PBMCs from 3 individuals (data not shown). These data suggest that SARS-CoV-2-specific T cell responses detected in expanded PBMCs from convalescent individuals were induced due to SARS-CoV-2 infection and not preexisting cross-reactive T cells or *de novo* induction of SARS-CoV-2-specific T cells. While the magnitude of T cell responses in expanded PBMCs may be biased due to differences in proliferation of each T cell clone during culture, *in vitro* expansion of antigen-specific T cells for comprehensive T cell analysis is often a necessary approach in clinical studies where blood volumes are extremely limited.

The breadth of T cell responses, which is a critical parameter in the quality of T cell immunity, was evaluated in S and N proteins, and memory T cells that recognize multiple regions across these proteins were maintained in convalescent individuals in the present study. Since robust T cell responses outside SARS-CoV-2 S and N antigens occur (5, 8, 9, 20, 41), the breadth of SARS-CoV-2-specific T cells recognizing the entire viral proteome in each individual could be considerably greater in COVID-19-convalescent individuals.

Tarke et al. performed comprehensive SARS-CoV-2 T cell epitope mapping using clinical samples collected in the United States and defined the immunodominant regions in S, N, and M proteins (9). Peptides frequently targeted in the present study only partially overlapped with immunodominant regions in the study by Tarke et al. (9), which may be due to different HLA allele frequency distributions between U.S. and Japanese populations. The strongest CD8^+^ T cell response observed in this study was against 301/302 OLP pairs (QELGKYEQYIKWPWYIWLG) located in the C terminus of S protein with the highest frequency. All 10 individuals with CD8^+^ T cell responses against this OLP pair possessed HLA-A*24:02. HLA- A*24:02 is the most prevalent HLA in the Japanese population (allele frequency, 0.361, compared to 0.065 in the North American study by Tarke et al. [9]). S protein peptide pair 301/302 has 5 predicted epitopes, of which 4 are HLA-A*24:02 restricted: KYEQYIKW, QYIKWPWYI, QYIKWPWYIW, and QYIKWPWYWL. Epitope QYIKWPWYI has been reported as an immunodominant HLA-A*24:02-restricted epitope (42, 43). Considering the different global distribution of HLA genotypes, evaluation of T cell responses should be assessed in each population based on genetic background.

Individuals with severe symptoms maintained T cell responses against multiple SARS-CoV-2 antigens following recovery, as previously described (5, 44–46). Patients with severe COVID-19 have higher viral loads and longer durations of virus shedding than those with mild symptoms (47, 48), so exposure to higher viral antigen for longer periods in severe cases may elicit stronger T cell responses. Notably, the breadth and magnitude of S protein-specific, but not N protein-specific, T cells were predominant in the severe group. The relative ratio of N to S protein in the breadth of the specific memory T cell responses was higher in individuals with mild symptoms, suggesting different contributions of S protein- and N protein-specific T cells in COVID-19 control. The relatively higher breadth of N to S protein-specific T cells may be associated with milder disease symptoms.

Since February 2022, the Omicron variant has represented over 98% of publicly available SARS-CoV-2 sequences. New Omicron subvariants are continuously emerging with accumulating amino acid mutations (38, 49, 50). These emerging viruses have shown decreased susceptibility to neutralization (18, 38, 50, 51), implying that escape from neutralizing antibodies is a driving force for viral evolution. However, the binding affinity to HLA class I molecules was preserved in 94 to 96% of CD8^+^ T cell epitopes located in regions of S and N proteins frequently targeted by T cells in our cohort, suggesting that amino acid mutations in the present SARS-CoV-2 variants have no serious impact on T cell immunity, similar to previous reports (34, 52–55). Limitations of our study include not having data on HLA-restricted epitopes for T cell responses in these frequently targeted regions and that T cell responses against mutant peptides were not assessed. In order to reveal the impact of amino acid changes in SARS-CoV-2 variants on T cell responses, more detailed analyses are required.

Some studies support the notion that escape mutations from T cell responses are accumulating in emerging SARS-CoV-2 variants (54, 56, 57). Amino acid changes in T cell target peptides should continue to be carefully monitored in emerging viruses. Identification of T cell target regions at the individual level and evaluation of immunodominant regions at the population level are essential for an accurate understanding of the impact of amino acid mutations on SARS-CoV-2 transmission and pathogenesis.

## MATERIALS AND METHODS

### Study population.

Volunteers over the age of 18 years who recovered from COVID-19 were recruited via the Internet. All applicants received an in-person explanation, and informed consent was obtained from all participants prior to the conduct of study procedures. Thirty-eight convalescent individuals, who were infected between March 2020 and January 2021, participated in the study. All participants were Japanese. SARS-CoV-2 infection was confirmed by PCR-based testing at hospitals or clinics. Characteristics of participants are shown in Table 1. Individuals were divided into two groups, severe and mild, based on pneumonia diagnosed by chest computed tomography or X-ray during COVID-19 (Table 1). Three participants in the severe group received oxygen. This study was approved by ethics committees of the Japan Conference of Clinical Research (no. 384) and the National Institute of Infectious Diseases (no. 1192).

### Human leukocyte antigen genotyping.

Genomic DNA was extracted from PBMCs using the QIAamp DNA blood minikit (Qiagen). HLA genotyping was performed with the AlloSeq Tx17 kit (CareDx) following the manufacturer’s instructions. Next-generation sequencing (NGS) libraries were sequenced by the MiSeq (Illumina) instrument. Bioinformatics analysis was done with the bioinformatics pipeline AlloSeq Assign (CareDx).

### Peptides.

Pools of 15-mer peptides (JPT Peptide Technologies), overlapping by 11 amino acids and spanning the full proteome of SARS-CoV-2, with the exception of ORF1ab, were used as SARS-CoV-2 antigens. Overlapping peptide (OLP) pools of S, N, M, and envelope (E) proteins, ORF3a, ORF6, ORF7a, ORF7b, ORF8, ORF9b, and ORF10 with amino acid sequences representing wild-type SARS-CoV-2 (Wuhan strain) were composed of 315, 102, 53, 16, 66, 13, 28, 8, 28, 22, and 7 OLPs, respectively.

Additionally, OLPs included in the S and N protein OLP pools were synthesized individually (Eurofin Genomics) to facilitate comprehensive screening as described previously (58). All 315 and 102 OLPs covering the entire S and N proteins, respectively, were included in a peptide matrix for each protein. To reduce the number of PBMCs required for analysis, adjacent OLPs were paired (see Fig. S3 in the supplemental material), resulting in 157 and 51 OLP pairs for the S and N protein matrices, respectively. Each OLP pair was represented in two different pools, allowing identification of the respective peptides in two corresponding pools, with maxima of 26 peptides for the S protein and 16 for the N protein pools.

### *In vitro* expansion of PBMCs.

PBMCs were stimulated with 100 ng/mL of OLP pools of SARS-CoV-2 proteins and cultured for 2 weeks in RPMI 1640 supplemented with 10% heat-inactivated fetal calf serum, 100 U/mL of penicillin, 100 μg/mL of streptomycin, and 2 mM glutamine with 50 U/mL of IL-2 (Miltenyi Biotec). Expanded PBMCs were cryopreserved prior to use.

### IFN-γ ELISpot assay.

The interferon gamma (IFN-γ) ELISpot assay was performed on cryopreserved samples as previously described (58). One hundred thousand *ex vivo* or expanded PBMCs were cultured overnight with peptides. The final concentration of each peptide for the ELISpot assay with OLP pools for each protein was 100 ng/mL.

Two-dimensional peptide matrices were designed for screening of T cell target peptides by the ELISpot assay using expanded PBMCs. The final concentration of each peptide was 2 μg/mL. All peptides located at the intersection of positive row and column wells in the matrices were tested individually to confirm responses.

Thresholds for positive responses were determined as at least 30 and 50 spot-forming cells (SFC)/10^6^ cells for PBMCs and expanded PBMCs, respectively, and/or responses exceeding two times the mean negative (medium-only) wells, whichever was greater. Results are reported as SFC/10^6^ PBMCs.

### ICS.

Intracellular cytokine staining (ICS) was performed as previously described (58). Expanded PBMCs from each participant were stimulated with the reactive OLP pairs at 10 μg/mL for 6 h in the presence of anti-CD28, anti-CD49d, and an inhibitor of the Golgi transporter.

The following antibodies and reagents were used for ICS: anti-CD4-fluorescein isothiocyanate (FITC), anti-CD8-peridinin chlorophyll protein (PerCP), anti-CD3-pacific blue, anti-tumor necrosis factor alpha (TNF-α)–allophycocyanin (APC), anti-IFN-γ–APC–Cy7 (BioLegend), anti-CD28, anti-CD49d, GolgiStop (BD Biosciences), and LIVE/DEAD fixable aqua dead cell stain kit (Thermo Scientific). Flow data were acquired using a FACS Canto II (BD Biosciences) and analyzed using FlowJo software version 10.8.1 (Tree Star). T cell responses were defined as positive if the frequency of both IFN-γ- and TNF-positive cells was more than 0.1% in total CD3^+^ CD4^+^ or CD3^+^ CD8^+^ T cells.

### T cell epitope prediction.

T cell epitope prediction was performed using the NetMHCpan-4.1 web server (40) for HLA-A, -B, and -C, for allele frequencies greater than 1% in the Japanese population. Peptides with a percent rank of <0.5 were defined as predicted epitopes following the recommendation of the software developer.

### Statistical analysis.

GraphPad Prism 8 (GraphPad Software, San Diego, CA, USA) was used for all statistical analyses. The paired *t* test was used for comparisons between T cell subset responses, and the Mann-Whitney test was used for all other comparisons. Correlations were performed with Spearman’s rank correlation.
