# Supplementary figures and images for: Breadth and Durability of SARS-CoV-2-Specific T Cell Responses following Long-Term Recovery from COVID-19
Source: Microbiol Spectr. 2023 Jul 10;11(4):e02143-23. doi: 10.1128/spectrum.02143-23 (PMC10433967; doi:10.1128/spectrum.02143-23)

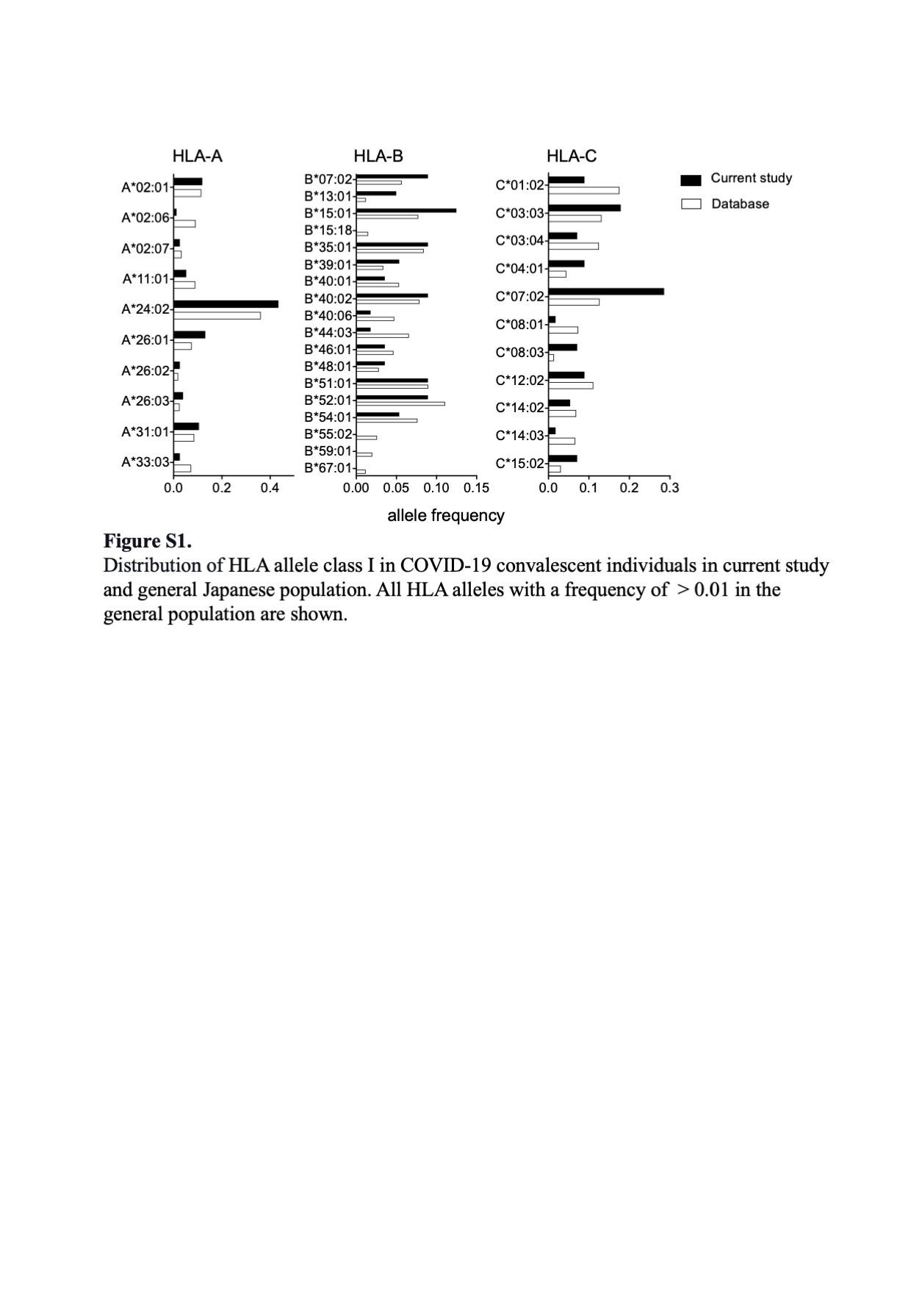

Supplement: Supplemental file 1 — Fig. S1. Download spectrum.02143-23-s0001.tif, TIF file, 6.4 MB [file spectrum.02143-23-s0001.tif]

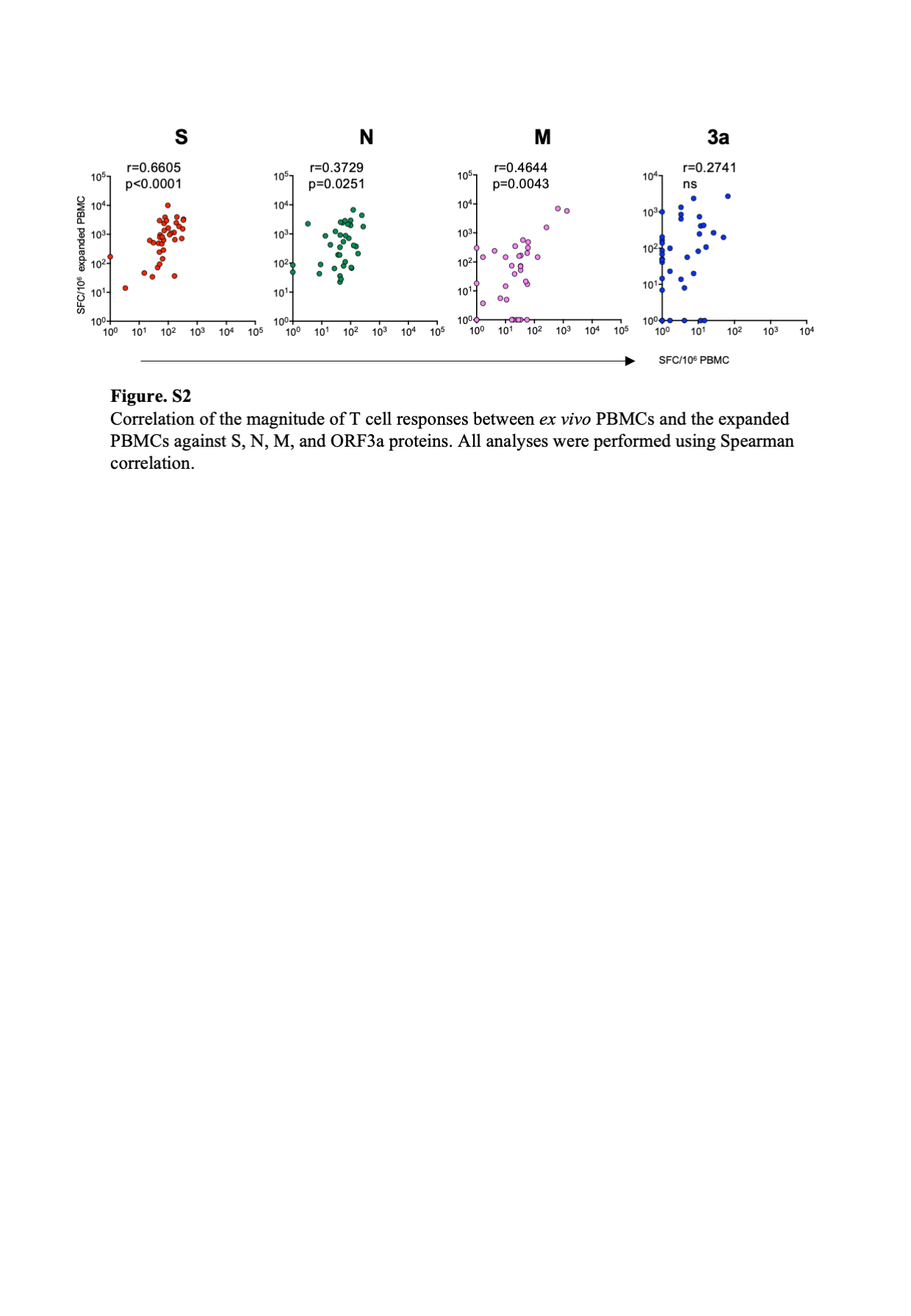

Supplement: Supplemental file 2 — Fig. S2. Download spectrum.02143-23-s0002.tif, TIF file, 6.4 MB [file spectrum.02143-23-s0002.tif]

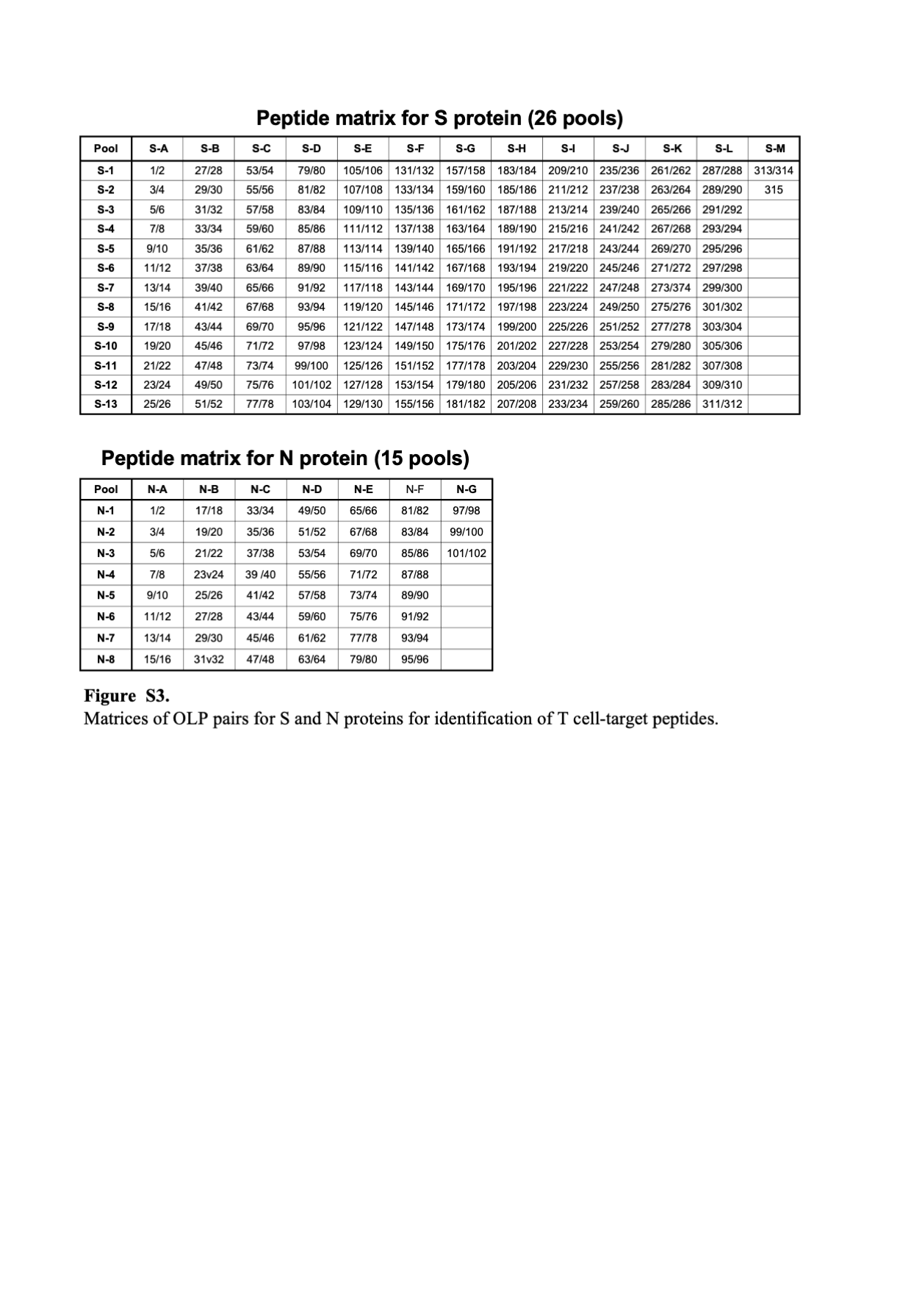

Supplement: Supplemental file 3 — Fig. S3. Download spectrum.02143-23-s0003.tif, TIF file, 6.4 MB [file spectrum.02143-23-s0003.tif]

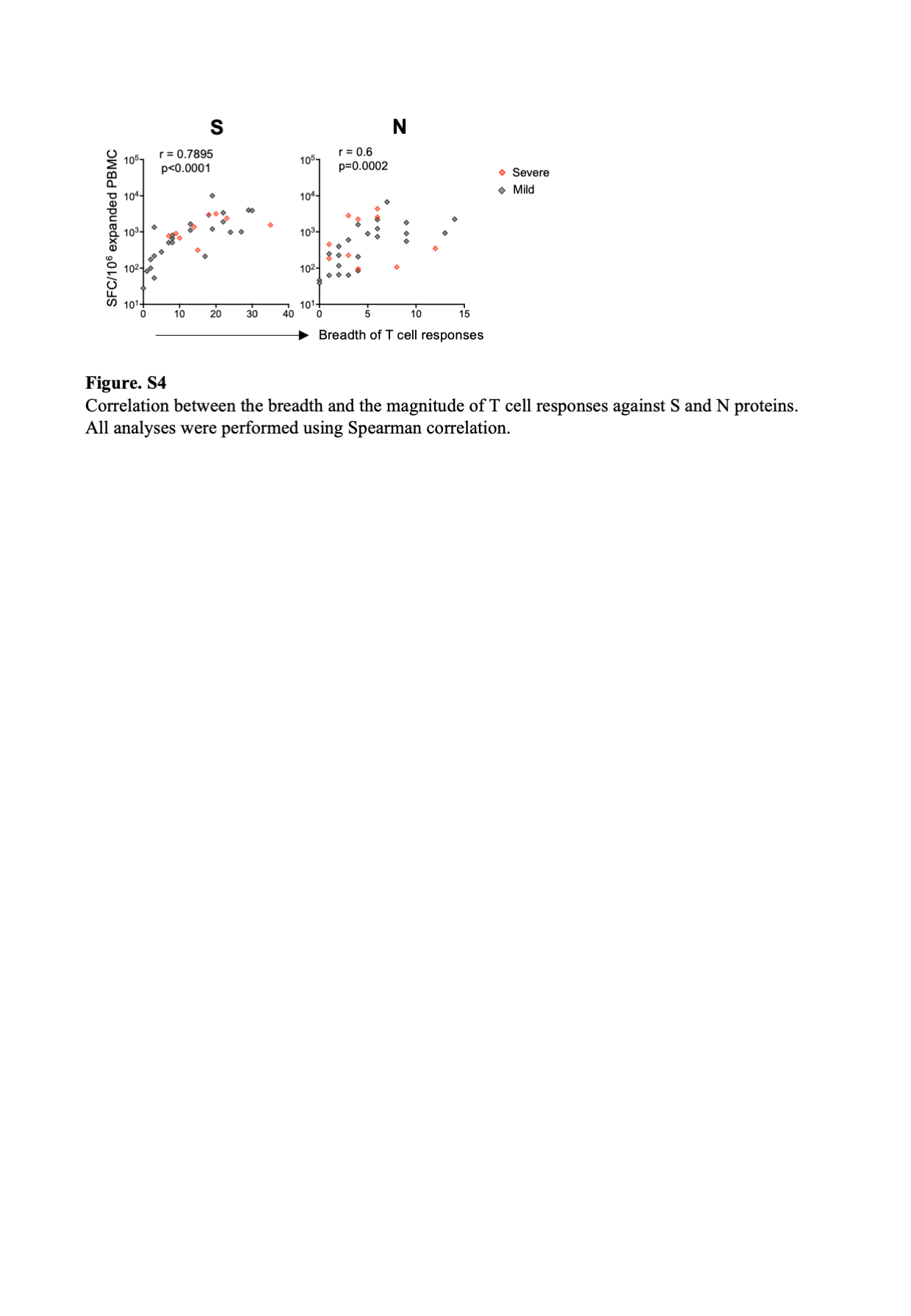

Supplement: Supplemental file 4 — Fig. S4. Download spectrum.02143-23-s0004.tif, TIF file, 6.4 MB [file spectrum.02143-23-s0004.tif]
